# Recent Trends of Metabolic Syndrome and Its Components in Military Recruits from Saudi Arabia

**DOI:** 10.3390/medicines8110065

**Published:** 2021-10-30

**Authors:** Hamoud Abdullah Al-Shehri, Abdulrahman Khazim Al-Asmari, Haseeb Ahmad Khan, Ghaleb Bin Horaib, Ahmed Al-Buraidi, Abdullah Ali Al-Sharif, Saeed Ghander Kadasah, Saud Al-Omani, Fayez S. Mohammed, Rajamohamed Abbasmanthiri, Nasreddien Mohammed Osman

**Affiliations:** 1 Adult Cardiology, Prince Sultan Cardiac Center, Medical Service Department (MSD), Ministry of Defence, Riyadh 11159, Saudi Arabia; drmoshabek@hotmail.com; 2 Scientific Research Center, Medical Service Department (MSD), Ministry of Defence, Riyadh 11159, Saudi Arabia; amohamed@psmmc.med.sa (R.A.); nmaosman.1@gmail.com (N.M.O.); 3 Department of Biochemistry, College of Science, King Saud University, Riyadh 11451, Saudi Arabia; haseeb@ksu.edu.sa; 4 Dermatology Department, Medical Service Department (MSD), Ministry of Defence, Riyadh 11159, Saudi Arabia; ghoraib@msd.med.sa; 5 Department of ENT, Prince Sultan Military Medical City, Medical Service Department (MSD), Ministry of Defence, Riyadh 11159, Saudi Arabia; aalburaidi36@hotmail.com; 6 Department of Dentistry, Prince Sultan Military Medical City, Medical Service Department (MSD), Ministry of Defence, Riyadh 11175, Saudi Arabia; drendo2001@yahoo.com; 7 Department of Psychiatry, Prince Sultan Military Medical City, Medical Service Department (MSD), Ministry of Defence, Riyadh 11159, Saudi Arabia; skadasah@yahoo.com; 8 Department of Surgery, Prince Sultan Military Medical City, Medical Service Department (MSD), Ministry of Defence, Riyadh 11159, Saudi Arabia; saudomani@hotmail.com; 9 Prince Sultan Military College of Health Science, Dhahran 34315, Saudi Arabia; Fa2.Fa2@hotmail.com

**Keywords:** metabolic syndrome, military recruits, prevalence, lipid profile, blood pressure, blood glucose, body weight

## Abstract

Metabolic syndrome (Met-S) constitutes the risk factors and abnormalities that markedly increase the probability of developing diabetes and coronary heart disease. An early detection of Met-S, its components and risk factors can be of great help in preventing or controlling its adverse consequences. The aim of the study was to determine the prevalence of cardio-metabolic risk factors in young army recruits from Saudi Arabia. A total of 2010 Saudis aged 18–30 years were randomly selected from groups who had applied to military colleges. In addition to designed questionnaire, anthropometric measurements and blood samples were collected to measure Met-S components according to the International Diabetes Federation (IDF) criteria. Met-S prevalence was 24.3% and it was higher in older subjects than the younger ones. There were significant associations between Met-S and age, education level and marital status. The most common Met-S components were high fasting blood sugar (63.6%) followed by high blood pressure (systolic and diastolic, 63.3% and 37.3% respectively) and high body mass index (57.5%). The prevalence of pre-diabetes and diabetes were found to be 55.2% and 8.4%, respectively. Hypertriglyceridemia was found in 19.3% and low levels of high-density lipoproteins (HDL) in 11.7% of subjects. In conclusion, there is a high prevalence of Met-S in young adults of Saudi Arabia. There is a need for regular monitoring of Met-S in young populations to keep them healthy and fit for nation building. It is also important to design and launch community-based programs for educating people about the importance of physical activity, cessation of smoking and eating healthy diet in prevention of chronic diseases.

## 1. Introduction

Metabolic syndrome (Met-S) is defined as a cluster of cardiovascular disease (CVD) risk factors that typically include central obesity, elevated blood pressure (BP), impaired glucose metabolism, and dyslipidemia. Met-S is a major risk factor for cardiovascular disease [1] and type 2 diabetes [2]. Since the turn of the 21st century, Saudi Arabia has witnessed marked socio-economic developments in health, education, environment, urban migration, and lifestyle. These developments have led to a decrease in communicable diseases, but an increase in chronic diseases of lifestyle, such as obesity, diabetes, hypertension and other risk factors of CVD [3]. The CVD risk factors include behavioural (smoking, diet, physical activity, alcohol consumption), physiological (blood cholesterol, hypertension, blood glucose, BMI), and metabolic disorders [4]. A low socio-economic position and an inadequate nutrient intake are also associated with an elevated CVD risk [5,6].

Soldiers are typically young and physically active but they are not devoid of CVD risk when they are overweight or obese [7,8]. Soldiers generally have a low incidence of CVD risk factors, which can be explained by regular exercise, high aerobic fitness and/or heredity factors. However, the globally increasing overweight and obesity trends are also emerging in military populations. Some studies have reported an increasing trend of cardiovascular risk factors among military personnel [9,10]. However, fewer studies have quantified the prevalence of undiagnosed hypertension and overweight/obesity among military personnel. Al-Asmary et al. [11] reported the prevalence of undiagnosed hypertension 17.53% and a combined prevalence of overweight/obesity of 66.8% in a community-based screening among military personnel from Saudi Arabia. In Brazil, the prevalence of overweight/obesity among young military personnel was estimated to be 36% [12]. A study from Sudan reported the prevalence of undiagnosed hypertension and overweight/obesity as 69.9% and 49.2%, respectively, among the police forces [13]. Overweight and obese soldiers are at a higher risk of elevated levels of serum lipids and other CVD risk factors [8,14].

Military personnel as an occupational group are at higher risk of stressful conditions, exposure to death or harmful agents as well as imposed restriction on food selection or availability. Having an ideal body weight and fitness is a fundamental principle in military forces recruitment. Unfortunately, there are scarce data about the prevalence of CVD risk factors and almost no research on the prevalence of Met-S in young army recruits from Saudi Arabia. We therefore examined the prevalence of Met-S and associated risk factors in army recruits who are expected to have a better fitness level than the general population.

## 2. Materials and Methods

A total of 2010 young Saudi men aged 18–30 years who applied for recruitment to Saudi armed forces were included in this study. An estimated 4000 applicants are annually invited to participate in the recruitment process. The power analysis based on a previous prevalence study in general Saudi population [15] and using 95% confidence interval and a precision of 5% showed that a sample size of 1748 subjects would satisfy the statistical requirement. We slightly increased the sample size because our target samples were young adults. We conducted the sampling form in multiple recruitment centres until the target sample size was achieved. The study was carried out at the health facilities of the selected centres and all the selected participants individually completed a consent form. Standardized medical observations included physical examination as well as measurements related to Met-S including blood pressure, waist circumference, height, body weight and blood biochemistry. Biochemical parameters included blood glucose and lipid profile. The complete information of each participant was filled in using a specially designed questionnaire based on the guidelines of World Health Organization [3]. The study protocol was approved by Institutional Review Board.

According to the International Diabetes Federation (IDF) definition, subjects were considered to have Met-S if they had central obesity (defined as waist circumference > 94 cm), plus two of the following four factors: raised fasting plasma glucose > 100 mg/dL (5.6 mmol/L), or previously diagnosed type 2 diabetes; systolic blood pressure (BP) > 130 or diastolic BP > 85 mm Hg, or treatment of previously diagnosed hypertension; high density lipoproteins (HDL) < 40 mg/dL (1.0 mmol/L) or specific treatment for this lipid abnormality; triglycerides (TG) level > 150 mg/dL (1.7 mmol/L) or specific treatment for this lipid abnormality.

Prehypertension was defined as systolic blood pressure 120–139 mm Hg or diastolic blood pressure 80–89 mm Hg. Hypertension was defined as systolic blood pressure ≥ 140 mm Hg or diastolic blood pressure ≥ 90 mm Hg [16]. BMI was classified according to the WHO adult BMI classification into normal (18.50–24.99 kg/m^2^), overweight (≥25–29.99 kg/m^2^) and obese (≥30 kg/m^2^) (Global Database on Body Mass Index: BMI classification [3]). 

Blood samples from each recruitment centre were transported to Prince Sultan Military Medical City for biochemical analysis. Blood samples were centrifuged at 1500× *g* for 15 min, at 4 °C, and sera were stored for analysis. Fasting blood sugar (FBS), total cholesterol, HDL and TG were analysed using a Roche Hitachi 902 autoanalyzer (Roche, Mannheim, Germany). 

The data were analysed by using the SPSS statistical package version 14 (SPSS, Chicago, IL, USA). Mean and standard deviation (SD) were calculated for parametric data, while categorical data were represented by number and percentage. The chi-square test was used for comparison between the Met-S and without Met-S groups. A *p* value < 0.05 was considered as statistically significant. 

## 3. Results

The anthropometric and demographic characteristics as well as blood pressure and pulse rate are summarized in Table 1. The mean age of participants was 20.16 years, with a median of 19 years. Categorical distribution of demographic characteristics is summarized in Table 2. The majority of participants were below 22 years old (72%) while only 2.4% belonged to age category between 27–30 years. Regarding the education of participants, most of them (73.1%) took <15 years of school education and only 7 subjects (0.3%) attended the higher level of education (Table 2). There were very few married participants (3.8%). The monthly income of a large number of subjects was <20,000 Saudi Riyals (SR) per month (Table 2). 

The most striking finding of study was the high frequency of subjects with >30 kg/m^2^ BMI (46.2%) despite the young age of these participants. More than a quarter of total participants were underweight (28.6%), while only limited number of subjects (14.0%) had normal body weight (Table 3). One third of total participants had an abnormally large waist circumference. Although diastolic blood pressure was normal in 62.7% of subjects, systolic blood pressure was normal only in 36.7% of participants. The pulse rate was found to be high in the majority of subjects (Table 3).

The answers of subjects to queries about their awareness of hypertension and diabetes as well as any medication are summarized in Table 4. Regarding the measurement of blood pressure (BP), more than 90% subjects were unaware of their BP readings. Only 10 participants (0.5%) were receiving treatment for BP control; few of them were taking herbal medicine. More than 95% of subjects were never advised to reduce their salt intake, stop smoking, reduce weight or perform regular exercise. In reply to whether their blood sugar levels had been checked at a clinic, only 22.8% subjects were affirmative (Table 4). Out of total 2010 subjects, only 34 (1.7%) knew about their diabetes and only 3% of subjects were aware of their hyperglycemia during the last one year. None of the participants were taking insulin therapy, while only 1.5% subjects were receiving oral medications. Only a few participants had received advice about the role of healthy diet, maintaining normal body weight, abstaining smoking and regular exercise in controlling blood sugar levels (Table 4).

The results of serum biochemistry including fasting blood sugar (FBS) and lipid profile are given in Table 5. A large number of subjects (63.6%) showed high levels (>99 mg/dL) of fasting blood sugar (FBS). However, triglycerides (TGs) and HDL were found to be normal in 80.7% and 88.3% of participants (Table 6).

**Figure 1:**
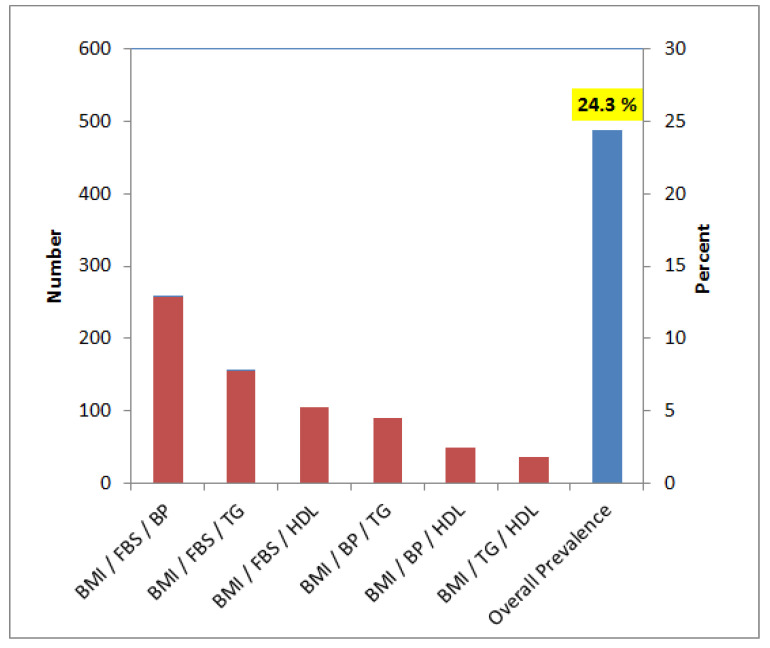
Prevalence of Met-S using various triplet components. Blue bar shows cumulative filter counts from all six triplets of Met-S components.

For the diagnosis of Met-S, six different triplets of associated components were used as a standard protocol. Specific cut-off values of these triplet parameters were used to filter subjects with Met-S. Among these triplets, BMI/FBS/BP identified 12.9% subjects affected with Met-S, followed by BMI/FBS/TG (7.8%), BMI/FBS/HDL (5.3%), BMI/BP/TG (4.5%), BMI/BP/HDL (2.5%) and BMI/TG/HDL (1.8%) (Table 7). The aggregate prevalence of Met-S was found to be 24.3% (Figure 1).

**Figure 2:**
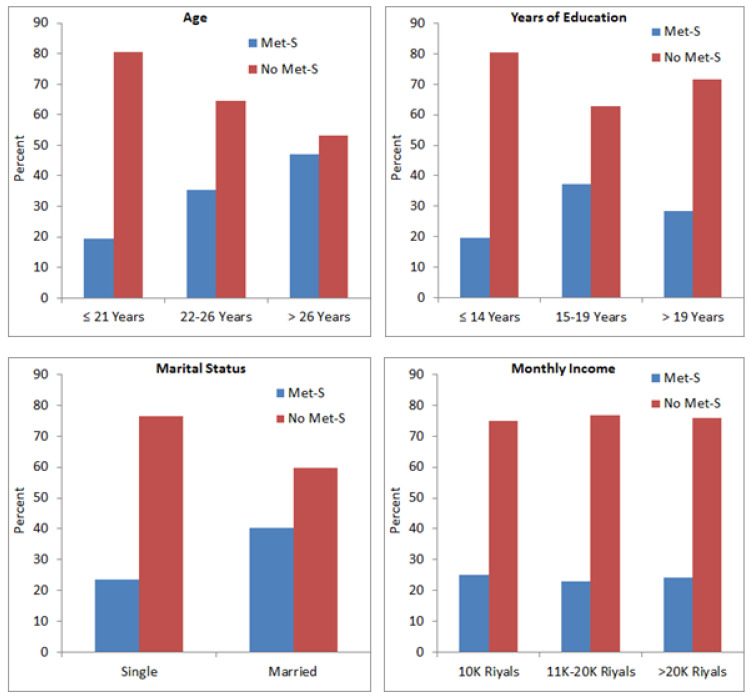
Association between Met-S and age, education, marital status and monthly income.

We observed significant association between Met-S and age (*p* = 0.001), education level (*p* = 0.001) and marital status (*p* = 0.003); however, monthly income (*p* = 0.761) was not associated with Met-S (Figure 2). There was a direct correlation between age and Met-S, as the subjects aged >26 years showed a high prevalence of Met-S compared to subjects ≤21 years. Subjects who received higher education showed comparatively higher frequency of Met-S than those who had received only school education (Figure 2). Married subjects showed significantly higher frequency of Met-S as compared to un-married subjects. The frequency of Met-S was almost the same in subjects with different income groups (Figure 2).

## 4. Discussion

This study was designed to measure the prevalence of metabolic syndrome (Met-S) and the associated risk factors in young men, as a sector in a population. In this study, Met-S was found in 24.3% of young adults (Figure 1). Worldwide, it is noted that the prevalence of Met-S is increasing [17,18,19], and according to International Diabetes Federation (IDF), approximately a quarter of the global population is estimated to have developed Met-S. A number of studies report different prevalence methods and criteria among different populations. Gyakobo et al. [20] reported an incidence of 35.9% using the IDF criteria whilst it was 15% using the National Cholesterol Education Program Adult Treatment Panel III (NCEP-ATP III). Regionally, Met-S was found to be 12% in United Arab Emirates (UAE) [21] as well as in young adults of Saudi Arabia [22]. Reports have shown a Met-S prevalence of 6.0% to 23.7% in the Gulf Cooperation Council (GCC) countries [23].

This study showed a significant association between age and Met-S (Figure 2). Demographic and anthropometric characteristics play important roles in the development of Met-S. A directly proportional increase of Met-S with age had been reported by several previous investigators [24,25]. Alexander et al. [26] suggested that fasting glucose, diabetes and systolic blood pressure (SBP) had a direct relationship with age and BMI, and that prevalence of diabetes and hypertension (components of Met-S) increased with age which also increased the incidence of Met-S. Other investigators also showed alarming rates of some risk factors that increased with age in cases of DM, HTN and BMI and they attributed this to high levels of blood sugar attained in their older ages due to inadequate involvement of individuals in physical activities [27,28].

We observed that a large number of participants were overweight. Obesity continues to rise in Arab population with an alarming rate, while females are more prone to becoming obese than males. Obesity is a progressively significant public health problem and is considered a major risk factor for diet-related chronic diseases including Met-S, T2DM, hypertension, stroke and certain forms of cancer [29]. Abdominal or central obesity remains one of the major clinical features of Met-S. Due to the high occurrence and complications associated with Met-S, a thorough understanding of the risk factors involved is vital to developing appropriate primary and secondary preventive measures [30].

In our study, mean pulse rate was found to be 84.77 ± 13.97 per minute and most of the subjects had a pulse rate above 72 beats/min. Al-Qurashi et al. [31] reported age-related variation in heart rate of Saudi children and adolescents. Heart rate variability (HRV, a measure of fitness) was significantly less in obese compared with normal weight Saudi male university students [32]. Heart rate variability was also found to be affected by short-term high-intensity interval training versus moderate-intensity continuous training in physically inactive adults [33]. Alkahtani et al. [34] investigated the effect of recreational aerobic physical activity type and volume on HRV in Arab men. They concluded that walking > 150 km per month or cycling > 100 km per month at a speed > 20 km/h may be necessary to derive cardiac autonomic benefits from physical activity among Arab men. Alassiri et al. [35] reported that keeping cell phones in a chest pocket reduced the HRV of normal-weight and obese medical students and exaggerated the effect of obesity on sympathetic activation. In a case-control study on 101 adult males, long-term use of dipping tobacco was not associated with permanent changes in heart rate and blood pressure whereas acute tobacco dipping caused an acute increase in heart rate [36]. Chocolate consumption was not correlated with heart rate variability among young adult participants [37].

The majority of our subjects had fasting blood sugar (FBS) greater than 99 mg/dL. A nationwide, population-based cohort of 53,370 participants from Saudi Arabia revealed that abnormal glucose metabolism has reached an epidemic state in Saudi Arabia, where one-third of the population is affected and half of diabetic cases were unaware of their disease [38]. It is important to note that prediabetes condition in young Saudi adults is associated with dyslipidemia, reduced total antioxidant status, obesity and physical inactivity compared to those with normoglycemia [39]. In a recent study, prevalence of uncontrolled FBS has been reported to be high among Saudi diabetic patients while risk factors associated with uncontrolled FBS include older age, male gender, hypertension, smoking and obesity. Moreover, uncontrolled hyperglycemia has also been directly associated with dyslipidemia [40,41].

Military personnel are considered healthy and physically fit adults who may be at low risk for developing a cardio-metabolic disease, as military service requires adherence to body composition, fitness and medical standards [42]. However, evidence suggests that biomarkers and health-risk behaviours associated with Met-S in military personnel may be similar to that observed in civilians [38,43,44]. Compared to previous reports [45], military recruits are now less physically fit and are larger, with elevated body fat, highlighting the necessity for regular surveys, monitoring and effective primary prevention strategies. Although Saudi civilians and soldiers show a high increase in Met-S, information on the nature and level of the contributory risk factors of Met-S among young Saudi adults is scarce.

In conclusion, the prevalence of Met-S was found to be 24.3% of young participants in this study, which is a matter of concern. As we also observed a direct association between age and Met-S, it is expected that the prevalence of Met-S in older populations would be much higher. Elevated FBG was the most prevalent component of Met-S which might be indicative of the first detectable Met-S component factor in several adults. Blood pressure and BMI would also help in the screening of Met-S. It is known that long-term Met-S results in numerous complications; however, Met-S is preventable and controllable with lifestyle changes. Regular screening, follow-up studies and interventions would reduce the incidence of Met-S and improve the fitness of youngsters. The findings of this study serve as the latest survey of Met-S status in a young Saudi population. The results will help in designing preventive measures as well as public awareness programs for minimizing the incidence of Met-S and its associated diseases.

## Figures and Tables

**Table 1:** Anthropometric and demographic characteristics of study subjects.

Variable	Mean ± SD	Median [Range]	95% Confidence Interval
Age (years)	20.16 ± 2.93	19.0 [18.0–30.0]	20.03–20.29
Height (cm)	169.38 ± 6.88	169.0 [136.5–192.0]	169.08–169.68
Weight (kg)	75.26 ± 23.94	74.0 [35.0–138.0]	74.21–76.31
Education (years of study)	13.27 ± 12.0	12.0 [9.0–22.0]	13.18–13.37
BMI (kg/m^2^)	26.10 ± 7.49	28.00 [10.0–45.0]	25.77–26.43
Waist Ab (cm)	65.72 ± 29.35	67.0 [10.0–130.0]	76.24–79.19
Systolic blood pressure (mm/Hg)	133.93 ± 13.29	134.00 [93–202]	133.37–134.53
Diastolic blood pressure (mm/Hg)	79.89 ± 12.31	79.0 [43–162]	79.37–80.45
Pulse rate (counts/min)	84.77 ± 13.97	84.0 [44–137]	84.18–85.40

**Table 2:** Categorical distribution of demographic characteristics.

	Frequency (*N*)	Percent (%)
**Age**		
≤21.0	1448	72.0
22.0–26.0	513	25.5
27.0–30.0	49	2.4
Total	2010	100.0
**Education**		
≤14.0 (First period of education)	1470	73.1
15.0–19.0 (Second period of education)	533	26.5
20.0+ (Third, longest period of education)	7	0.3
Total	2010	100.0
**Marital status**		
Married	77	3.8
Single	1932	96.1
Divorced	1	0.1
Total	2010	100.0
**Family members > 18y**		
≤5 (Usual family)	1259	62.6
6–10 (Slightly large family)	588	29.3
11–15 (Medium large family)	141	7.0
16+ (Large family)	22	1.1
Total	2010	100.0
**Monthly income (Saudi Riyals)**		
10,000 (Low)	977	48.6
11,000–20,000 (Medium)	843	41.9
>20,000 (High)	137	6.8
Refused to answer	53	2.6
Total	2010	100.0

**Table 3:** Categorical distribution of anthropometric characteristics.

	Frequency (*N*)	Percent (%)
**BMI**		
≤18.4 (Underweight)	574	28.6
18.5–24.9 (Normal weight)	281	14.0
25.0–29.9 (Overweight)	226	11.2
>30.0 (Obese)	929	46.2
Total	2010	100.0
**Waist circumference**		
Normal (≤94 cm)	1514	75.3
Abnormal (>94 cm)	496	24.7
Total	2010	100.0
**Blood pressure (systolic)**		
≤129.0 (Normal)	737	36.7
>129.0 (Abnormal)	1273	63.3
Total	2010	100.0
**Blood pressure (diastolic)**		
≤84.0 (Normal)	1261	62.7
>84.0 (Abnormal)	749	37.3
Total	2010	100.0
**Pulse/min**		
≤72 (Normal)	378	18.8
>72 (High)	1632	81.2
Total	2010	100.0

**Table 4:** Queries about hypertension, diabetes and their treatment.

	‘Yes’ Answer	
	Frequency (*N*)	Percent (%)
**Hypertension Queries**		
Was BP checked by medical professional	497	24.7
Informed of BP	117	5.8
Informed of BP last 12 months	127	6.3
**Hypertension Treatment Queries**		
Any treatment for blood pressure (BP) at present	10	0.5
Medicine being used for last 2 weeks	13	0.6
Advised to reduce salt intake	43	2.1
Advised/treated to reduce body weight	58	2.9
Advised/treated to stop smoking	48	2.4
Advised to start/do more exercise	64	3.2
Herbal medicine for BP control	3	0.1
**Diabetes Queries**		
Blood sugar checked at clinic	458	22.8
Informed that you have diabetes	34	1.7
Hyperglycemia during last 12 months	61	3.0
**Diabetes Treatment Queries**		
Currently receiving treatment	28	1.4
Insulin	0	0.0
Oral medication	31	1.5
Prescribed diet	32	1.6
Advised/treated to reduce body weight	13	0.6
Advised/treated to stop smoking	13	0.6
Advised to start/do more exercise	15	0.7
Herbal drugs for diabetes control	3	0.1

**Table 5:** Serum biochemistry of study subjects.

Variable	Mean ± SD	Median [Range]	95% Confidence Interval
Fasting blood glucose (mg/dL)	105.46 ± 16.77	104.0 [65–232]	104.73–106.20
Total cholesterol (mg/dL)	190.63 ± 57.84	185.70 [58.7–448.4]	188.10–193.16
High density lipproteins (HDL) (mg/dL)	66.53 ± 35.72	58.00 [7.0–298.0]	64.97–68.09
Triglycerides (mg/dL)	110.18 ± 61.02	99.00 [12.0–457.0]	107.51–112.85

**Table 6:** Categorical distribution of biochemical parameters.

	Frequency (*N*)	Percent (%)
**Fasting Blood sugar (FBS)**		
≤99 mg/dL (Normal fasting blood sugar)	731	36.4
>99 mg/dL (Abnormal fasting blood sugar)	1279	63.6
Total	2010	100.0
**Triglycerides (TGs)**		
≤149 mg/dL (Normal TGs)	1622	80.7
>149 mg/dL (High TGs)	388	19.3
Total	2010	100.0
**High Density Lipoproteins (HDL)**		
≤40 mg/dL (Low)	235	11.7
>40 mg/dL (Normal)	1775	88.3
Total	2010	100.0

**Table 7:** Diagnosis of Met-S using the triplets of Met-S component factors.

Triplets	Met-S Components	With High Cut-Off, *N*	Percent (%)	95% Confidence Interval
**Triplet 1 (BMI/FBS/BP)**	BMI	1114	55.4	53.2–57.5
	FBS	1279	63.6	61.5–65.7
	BPS/BPD	1262	62.8	60.6–64.8
	Filter count	260	12.9	11.4–14.4
**Triplet 2 (BMI/FBS/TGs)**	BMI	1114	55.4	53.2–57.5
	FBS	1279	63.6	61.5–65.7
	TGs	382	19.0	17.2–20.7
	Filter count	157	7.8	6.6–8.9
**Triplet 3 (BMI/FBS/HDL)**	BMI	1114	55.4	53.2–57.5
	FBS	1279	63.6	61.5–65.7
	HDL	254	12.6	11.2–14.1
	Filter count	106	5.3	4.3–6.2
**Triplet 4 (BMI/BP/TGs)**	BMI	1114	55.4	53.2–57.5
	BP	1262	62.8	60.6–64.8
	TGs	382	19.0	17.2–20.7
	Filter count	90	4.5	3.6–5.4
**Triplet 5 (BMI/BP/HDL)**	BMI	1114	55.4	53.2–57.5
	BP	1262	62.8	60.6–64.8
	HDL	254	12.6	11.2–14.1
	Filter count	50	2.5	1.8–3.2
**Triplet 6 (BMI/TGs/HDL)**	BMI	1114	55.4	53.2–57.5
	TGs	384	19.1	17.4–20.8
	HDL	254	12.6	11.2–14.1
	Filter count	36	1.8	1.2–2.3
**Total Met-S (Triplets 1–6)**	Cumulative filter count	488	24.3	22.4–26.1

Abbreviations are: Met-S, metabolic syndrome; BMI, body mass index, FBS, fasting blood sugar; BP, blood pressure; BPS, blood pressure systolic; BPD, blood pressure diastolic; TGs, triglycerides; HDL, high density lipoproteins cholesterol.

## Data Availability

Data requests should be addressed to corresponding author.
